# Receptor Mechanisms Mediating the Pro-Nociceptive Action of Hydrogen Sulfide in Rat Trigeminal Neurons and Meningeal Afferents

**DOI:** 10.3389/fncel.2017.00226

**Published:** 2017-07-27

**Authors:** Kseniya Koroleva, Alsu Mustafina, Aleksey Yakovlev, Anton Hermann, Rashid Giniatullin, Guzel Sitdikova

**Affiliations:** ^1^Department of Human and Animal Physiology, Institute of Fundamental Medicine and Biology, Kazan Federal University Kazan, Russia; ^2^Department of Cell Biology and Physiology, University of Salzburg Salzburg, Austria; ^3^A.I. Virtanen Institute for Molecular Sciences, University of Eastern Finland Kuopio, Finland

**Keywords:** pain, hydrogen sulfide, trigeminal nerve firing, trigeminal neurons, TRPV1-and TRPA1 receptors, Ca^2+^-imaging

## Abstract

Hydrogen sulfide (H_2_S), a well-established member of the gasotransmitter family, is involved in a variety of physiological functions, including pro-nociceptive action in the sensory system. Although several reports have shown that H_2_S activates sensory neurons, the molecular targets of H_2_S action in trigeminal (TG) nociception, implicated in migraine, remains controversial. In this study, using suction electrode recordings, we investigate the effect of the H_2_S donor, sodium hydrosulfide (NaHS), on nociceptive firing in rat meningeal TG nerve fibers. The effect of NaHS was also explored with patch-clamp and calcium imaging techniques on isolated TG neurons. NaHS dramatically increased the nociceptive firing in TG nerve fibers. This effect was abolished by the TRPV1 inhibitor capsazepine but was partially prevented by the TRPA1 blocker HC 030031. In a fraction of isolated TG neurons, NaHS transiently increased amplitude of capsaicin-induced currents. Moreover, NaHS by itself induced inward currents in sensory neurons, which were abolished by the TRPV1 inhibitor capsazepine suggesting involvement of TRPV1 receptors. In contrast, the inhibitor of TRPA1 receptors HC 030031 did not prevent the NaHS-induced currents. Imaging of a large population of TG neurons revealed that NaHS induced calcium transients in 41% of tested neurons. Interestingly, this effect of NaHS in some neurons was inhibited by the TRPV1 antagonist capsazepine whereas in others it was sensitive to the TRPA1 blocker HC 030031. Our data suggest that both TRPV1 and TRPA1 receptors play a role in the pro-nociceptive action of NaHS in peripheral TG nerve endings in meninges and in somas of TG neurons. We propose that activation of TRPV1 and TRPA1 receptors by H_2_S during neuro-inflammation conditions contributes to the nociceptive firing in primary afferents underlying migraine pain.

## Introduction

Hydrogen sulfide (H_2_S), a member of the gasotransmitter family along with nitric oxide (NO) and carbon monoxide, is involved in the regulation of great variety of physiological functions, including nociception and inflammation (Kawabata et al., 2007; Feng et al., 2013). The neuro-modulatory role of H_2_S was shown in the central and peripheral nervous system where it promotes the induction of long-term potentiation (LTP) in hippocampus (Abe and Kimura, 1996), inhibits giant depolarizing potentials in neonatal hippocampus (Yakovlev et al., 2017), affects NMDA-mediated currents (Abe and Kimura, 1996; Yakovlev et al., 2017), increases the transmitter release from motor nerve endings (Sitdikova et al., 2011; Gerasimova et al., 2013, 2015), or initiates contractile responses of the rat urinary bladder by stimulation of primary afferent neurons (Patacchini et al., 2004).

Increasing evidence suggests H_2_S to play a role in the emergence and conductance of somatic and visceral pain (Okubo et al., 2012). Intracolonical administration of sodium hydrosulfide (NaHS), a H_2_S donor, induced nociceptive behavior with abdominal hyperalgesia/allodynia (Matsunami et al., 2009). NaHS produced mechanical hyperalgesia in the rat hind paw in response to intraplantar administration (Kawabata et al., 2007). On the other hand, NaHS activates ATP-dependent K^+^ channels in different tissues (Tang et al., 2005; Mustafina et al., 2015) which may underlie the antinociceptive effects of NaHS (Distrutti et al., 2006).

Endogenously H_2_S is produced from L-cysteine by the enzymes cystathionine β-synthase (CBS), cystathionine γ-lyase (CSE) and 3-mercaptopyruvate sulfurtransferase along with additional contribution of cysteine aminotransferase or D-amino acid oxidase (Abe and Kimura, 1996; Renga, 2011). It was shown that CBS is widely expressed in rat trigeminal (TG) neurons (Feng et al., 2013) and its expression is upregulated in response to inflammatory pain with subsequent increase of the excitability of TG neurons by suppression of K^+^ conductance (Miao et al., 2014). It was reported that CBS is colocalized with transient receptor potential, vanilloid 1 (TRPV1) receptors in colon specific dorsal root ganglion (DRG) neurons (Xu et al., 2009; Qu et al., 2013). TRPV1 receptors undergo sensitization in response to inflammation which is mediated by the increased expression of CBS on DRG (Zhu et al., 2015).

Several recent publications indicated the ability of H_2_S to activate TRPV1 or TRPA1 receptors *in vitro* and *in vivo* experiments. Thus, the TRPV1 antagonist prevented NaHS-evoked luminal chloride secretion (Storti et al., 2015). NaHS-induced constriction of smooth muscle cells of airways and H_2_S-evoked intestinal motility were abolished by the TRPV1 antagonists (Trevisani et al., 2005; Bhatia et al., 2006). Moreover, NaHS increased the afferent neuronal activity in gut and induced inward currents in DRG neurons which were inhibited by TRPV1 antagonists (Lu et al., 2014).

However, there is also evidence indicating activation of TRPA1 receptors by H_2_S. Activation of capsaicin-sensitive sensory nerves through TRPA1 receptors by NaHS-induced vasodilatation resulting from the release of the vasoactive neuropeptides calcitonin gene-related peptide (CGRP) and substance P (Pozsgai et al., 2012; Hajna et al., 2016). Indirect evidence shows that activation of TRPA1 channels by H_2_S resulted in mechanical hyperalgesia and allodynia in mice (Okubo et al., 2012) whereas TRPA1 did not participate in pro-nociceptive effects of H_2_S in visceral tissues (Andersson et al., 2012). There is abundance evidence that H_2_S affects TRP channels in sensory neurons, but the molecular target of H_2_S action in nociceptive system remains to be determined.

The aim of this study was to explore the role of TRP receptors in the firing of TG nerve fibers induced by NaHS using extracellular recordings of peripheral branches of the TG nerve in isolated rat meninges and patch clamp recordings of TRPV1 currents as well as Ca^2+^-imaging of rat TG neurons.

## Materials and Methods

### Preparation and Solutions

All animal experiments were performed in accordance with the European Community Council Directive of September 22, 2010 (2010/63/EEC) and approved by the Animal Care and Use Committee of the University of Eastern Finland and the Ethics Committee of Kazan Federal University. Electrical activity of TG nerve was recorded using isolated hemiskull preparations obtained from adult (P35–36) rats as described previously (Shatillo et al., 2013). Firing activity was recorded from the *nervus spinosus* (V3 branch of the TG nerve) which was isolated and cleaned from the *dura mater*. This nerve innervates a region of the medial meningeal artery, supposed to initiate migraine pain and this model is widely used to investigate molecular mechanisms of migraine pain. The isolated preparation was washed for 20 min with Krebs solution containing (in mM): 120 NaCl; 2.5 KCl; 2 CaCl_2_; 1 MgCl_2_; 11 glucose; 1 NaHPO_4_; 24 NaHCO_3_ constantly gassed with 95% O_2_/5% CO_2_ and the pH kept at 7.2–7.4.

TG neurons were isolated from P9–P12 rats. Animals were anesthetized and decapitated. TG ganglia were excised and enzymatically dissociated in F12 medium containing 0.25 mg/ml trypsin, 1 mg/ml collagenase, and 0.2 mg/ml DNAase (Sigma) at 37°C. Cells were plated on poly-l-lysine-coated glasses in F12 medium with 10% fetal bovine serum and cultured for 1–2 days at 37°C in an atmosphere containing 5% CO_2_. During experiments cells were continuously perfused (at 2 ml/min) with a solution containing (in mM): 148 NaCl; 5 KCl; 1 MgCl_2_; 2 CaCl_2_; 10 HEPES; 10 D-Glucose; pH adjusted to 7.2 with NaOH. The intracellular solution for patch clamp experiments contained (in mM): 145 KCl; 2 MgCl_2_; 10 HEPES; 5 EGTA; 0.5 CaCl_2_; 2 Mg-ATP; 0.5 Na-GTP; 5 KCl; pH adjusted to 7.2 with KOH.

For Ca^2+^ imaging experiments cells were incubated for 40 min at 37°C in F12 medium supplemented with FBS 10% (Gibco Invitrogen, Carlsbad, CA, USA) containing fluo-3-AM (5 μM, Life Technologies, Foster City, CA, USA) followed by a 10–15 min washout period.

### Chemicals

Capsaicin, HC 030031 and capsazepine were dissolved in dimethyl sulfoxide (DMSO), dithiothreitol (DTT)—in external solution. DMSO in used concentration did not change the nociceptive activity (Zakharov et al., 2015). All substances were purchased from Sigma-Aldrich (St. Louis, MO, USA). NaHS (Sigma-Aldrich, St. Louis, MO, USA) was used as a source of H_2_S. In solution NaHS dissociates to give HS^−^ which associates with H^+^ to produce H_2_S. At 20°C—22.3% of total sulfide is present as H_2_S (Sitdikova et al., 2014). The real-time measurements of H_2_S in the chamber using amperometric sensors indicate a rapid loss of sulfide via H_2_S volatilization by bubbling with about 50% H_2_S loss within 3 min (Deleon et al., 2012; Sitdikova et al., 2014). In our experiments NaHS was used in a concentration of 100 μM which yields about 11 μM H_2_S in the perfusion system which constantly flows to the recording chamber. Stock solutions of NaHS were prepared immediately before each experiment and kept hermetically sealed in a dark place.

### Electrophysiology

TG nerve firing was recorded using a DAM 80 amplifier (band pass 0.001–3 kHz, gain 1000; World Precision Instruments, Sarasota, FL, USA). The *nervus spinosus* was placed inside the fire-polished glass recording microelectrode with a tip diameter of ~150 μm, filled with Krebs solution. A recovery period of at least 15 min was used to obtain stable baseline conditions. Control recordings of meningeal spikes were performed for 10 min previous to drug application. Signals were digitized at 125 kHz using a data acquisition board NI PCI6221 (National Instruments, Austin, TX, USA), and WinEDR software (Strathclyde University, Glasgow, UK). Five standard deviations (SD) were used to set the threshold for spike detection.

TRPV1 receptors are predominantly expressed in small- and medium-diameter neurons, which were used in our patch clamp experiments. TRPV1 currents were recorded at a holding potential of −70 mV using the whole-cell configuration of the patch clamp technique. TRPV1 currents were evoked by local application of capsaicin in a concentration of 1 μM for 2 s using a fast perfusion system (Rapid Solution Changer, RSC-200, BioLogic Science Instruments, Grenoble, France), with a solution exchange rate of ~20 ms. To prevent the desensitization of TRPV1 receptors, agonist was applied at intervals of 5 min. Responses to capsaicin were measured using a HEKA-10 amplifier and HEKA Patch Master Software (HEKA Electronic, Germany).

### Calcium Imaging

Fluorescence signals of neurons were recorded by microscope imaging setup (TILL Photonics GmbH, Munich, Germany) with a light excitation wavelength of 488 nm using respective filters. Images were collected in a time-lapse mode (500 ms exposure time) with a 12-bit CCD camera (SensiCam, Kelheim, Germany). Fluorescent signals from single cells were quantified as Δ*F*/*F*0, where *F*0 is the background subtracted baseline fluorescence and Δ*F* is the increment over baseline. The inhibitor of TRPV1—capsazepine (10 μM), the inhibitor of TRPA1—HC 030031 (50 μM), NaHS (100 μM, 2 s) and capsaicin (1 μM, 2 s) were applied via a fast perfusion system as indicated above, followed by application of a 50 mM KCl-containing solution to differentiate neurons. Data were analyzed off-line using Origin Pro 2015 (MicroCal, Northampton, MA, USA) software.

### Statistical Analysis

For each experiment we used at least three independent replicates (animals) and *n* means the number of cells. Normality of sample data was evaluated with Shapiro-Wilk test and for equal variances using *F*-test Origin Pro 2015 (OriginLab Corp., Northampton, MA, USA). Differences were considered as statistically significant at *p* < 0.05. All values are given as mean ± SEM. Statistical significance was determined by paired Student’s *t*-test and Mann-Whitney test.

## Results

### NaHS Increases Firing in Meningeal Nerve Terminals

**Figure 1:**
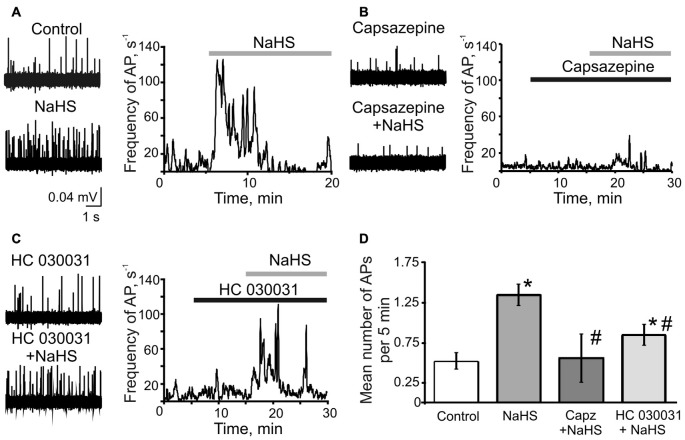
The role of TRPV1 and TRPA1 receptors in the effects of sodium hydrosulfide (NaHS) on trigeminal (TG) nerve firing.** (A)** Representative traces of TG nerve firing in meningeal hemiskull preparation (left panel) and frequency plots of action potentials (AP) vs. time during single experiments (right panel) in control and after application of NaHS (100 μM); **(B)** after inhibition of TRPV1 receptors by capsazepine (10 μM) and capsazepine + NaHS **(C)** after inhibition of TRPA1 receptors by HC 030031 (50 μM) and HC 030031 + NaHS. The solid bars indicate the application time of NaHS, capsazepine and HC 030031. **(D)** Plot of mean number of AP per 5 min during control, after application of NaHS, capsazepine (capz) + NaHS and HC 030031 + NaHS. *p < 0.05 compared to control; ^#^*p* < 0.05 compared to the effect of NaHS.

First, we tested if H_2_S can induce the nociceptive effect in meningeal nerves using a technique of suction electrode recording from meningeal TG nerve fibers (Zakharov et al., 2015). The peripheral part of the TG nerve (*nervus spinosus*) was placed inside the microelectrode and the orthodromic spontaneously generated action potentials (AP; spikes) generated at the periphery were recorded (Figure 1). Bath application of 100 μM NaHS induced a significant increase in the frequency of nociceptive spikes during the first 5 min to 320 ± 88% of control (control 0.53 ± 0.08 s^−1^, vs. 1.35 ± 0.13 s^−1^ in the presence of NaHS; *n* = 10, *p* < 0.0001; Figures 1A,D). During the next 5 min of application, the frequency of spikes did not differ significantly from control (0.95 ± 0.30 s^−1^, *p* = 0.2) and it returned to control level after 15 min of drug application (0.40 ± 0.01 s^−1^; *p* = 0.3; Figure 1A).

As previous studies suggested the action of NaHS in different tissues could be mediated either by TRPV1 or TRPA1 receptors (Trevisani et al., 2005; Andersson et al., 2012; Okubo et al., 2012; Lu et al., 2014; Hajna et al., 2016), we next examined the pro-nociceptive effect of NaHS in the presence of the specific inhibitors of these receptors. Inhibition of TRPV1 receptors by capsazepine (25 μM) did not significantly change TG nerve firing (0.40 ± 0.11 s^−1^ in control, vs. 0.32 ± 0.13 s^−1^ in capsazepine; *n* = 6, *p* = 0.14). Remarkably, the subsequent application of NaHS in the presence of capsazepine did not affect the frequency of nociceptive firing. The frequency of AP was 0.56 ± 0.30 s^−1^ (*p* = 0.5) during first 5 min of NaHS application, 0.44 ± 0.27 s^−1^ (*p* = 0.85) and 0.27 ± 0.15 s^−1^ during 10 and 15 min of application, respectively (*p* = 0.25; Figures 1B,D). The inhibition of TRPA1 by HC 030031 (50 μM) did not change firing itself (0.61 ± 0.11 s^−1^ in control, vs. 0.55 ± 0.09 s^−1^ in HC 030031; *n* = 7, *p* = 0.51), however, partially prevented the action of NaHS (increase to 161 ± 13%; 0.85 ± 0.13 s^−1^; *n* = 7, *p* = 0.007; Figures 1C,D). The NaHS effect after inhibition of TRPA1 was significantly lower than in control conditions. During 10 and 15 min of NaHS application the frequency of AP returned to the control level (0.70 ± 0.12 s^−1^, *p* = 0.13 and 0.82 ± 0.19 s^−1^, *p* = 0.27, respectively; Figure 1C). These results suggested the involvement of TRPV1 and TRPA1 receptors in the pro-nociceptive firing induced by NaHS in TG nerve fibers in meninges.

### NaHS Induces TRPV1 Mediated Currents in Isolated TG Neurons

**Figure 2:**
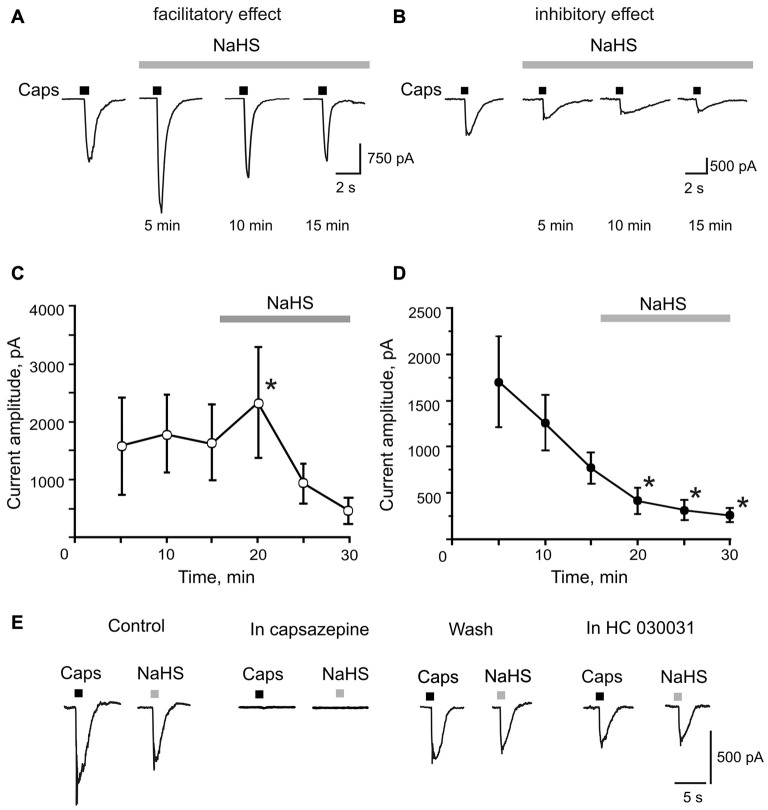
Facilitatory and inhibitory effects of NaHS on capsaicin induced currents in rat TG neurons. **(A,B)** Representative traces of capsaicin evoked currents (Caps, 1 μM, 2 s, short bars above traces) in control and during bath application of 100 μM NaHS for 15 min (solid bar above traces). Capsaicin was applied at an interval of 5 min to prevent desensitization of TRPV1 receptors. **(C,D)** Average amplitude of TRPV1 currents in control (three subsequent capsaicin application) and during NaHS application (solid bars above traces). Notice the increase of mean amplitude of TRPV1 currents after 5 min of NaHS perfusion in **(C)** and the constant decrease of capsaicin evoked currents in **(D)**. **(E)** Representative traces of currents evoked by focal application of 1 μM capsaicin (Caps, 2 s) and 100 μM NaHS (2 s) in control and after inhibition of TRPV1 receptors by capsazepine (10 μM); after washout and after inhibition of TRPA1 receptors by HC 030031 (50 μM). Notice that NaHS and capsaicin evoked currents were completely abolished by capsazepine. *p < 0.05 compared to the third application of capsaicin in control.

Next, we studied the effect of NaHS on TRPV1 receptors in single TG neurons using patch clamp recordings. The application of the TRPV1 receptor agonist capsaicin (1 μM, 2 s) induced inward currents in 22 out of 31 neurons with an average amplitude of 1345 ± 330 pA (*n* = 22; Figures 2A,B). In control, the repetitive application of capsaicin (interval 5 min) revealed two fractions of neurons: in one fraction, the amplitude of TRPV1 currents did not significantly change (*n* = 14, Figure 2C), whereas in another fraction rundown of currents was observed (*n* = 8, Figure 2D). These findings are consistent with an intrinsic heterogeneity of TRPV1 receptors with different rates of desensitization which was found in isolated sensory neurons previously (Akopian et al., 2007; Storti et al., 2015). We did not reveal differences in the membrane capacitance between two groups of TG neurons (36.9 ± 4.7 pF, *n* = 14 vs. 33.4 ± 7.7 pF, *n* = 8, *p* = 0.67). Superfusion with NaHS (100 μM) for 5 min revealed bidirectional effects on the fractions of cells with a different rate of desensitization. In a fraction of 14 cells with a low rate of desensitization, NaHS transiently increased currents from 1578 ± 501 pA to 2357 ± 715 pA (*n* = 14, *p* = 0.0065) followed by rundown during 10 and 15 min of NaHS application (Figures 2A,C). In other eight cells, we only observed a reduction of capsaicin-induced currents after 5 min exposure to NaHS from control value of 768 ± 168 pA to 414 ± 141 pA in the presence of NaHS (*n* = 8, *p* = 0.016; Figures 2B,D). In both cases, the effect of NaHS was not washable. The activating NaHS effects on TRPV1 currents could be explained by its reducing action on disulfide bonds of the TRPV1 channel protein (Susankova et al., 2006). Indeed, pre-application of 1 mM DTT prevented the increase of the current amplitude by NaHS. Thus, in the presence of DTT, NaHS decreased the amplitude of TRPV1 currents from 1360 ± 330 pA to 1025 ± 371 pA (*n* = 11, *p* = 0.0033) by 5 min of application with further reduction to 622 ± 171 (*p* < 0.0002) and then to 532 ± 167 pA (*p* < 0.0001), by 10 and 15 min, respectively (data not shown).

To investigate the direct effect of NaHS on TRPV1 currents, NaHS (100 μM) was applied through the fast perfusion system for 2 s. In cells responding to capsaicin, NaHS induced an inward current with an average amplitude of 413 ± 114 pA (*n* = 12; Figure 2E). Application of the TRPV1 receptor antagonist capsazepine (10 μM) completely blocked currents induced by NaHS and capsaicin (*n* = 5, Figure 2E). NaHS and capsaicin-induced currents were washable from the capsazepine inhibition. Subsequent application of the TRPA1 receptor selective antagonist HC 030031 (50 μM) on the same cell did not affect currents induced by NaHS and capsaicin (*n* = 5, Figure 2E). These data suggest that NaHS can directly activate TRPV1 receptors in rat TG neurons.

### NaHS Increases the Intracellular Ca^2+^ Concentration in Isolated TG Neurons

**Figure 3:**
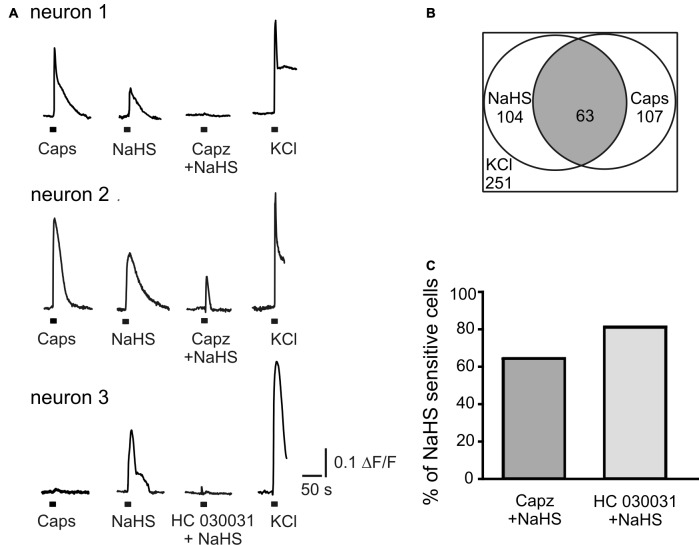
NaHS evokes calcium transients in TG neurons. **(A)** Representative traces recorded from cultured TG neurons. NaHS (100 μM) and capsaicin (Caps, 1 μM) were applied for 2 s to TG ganglion cells loaded with fluo 3-AM (5 μM). Neurons were distinguished from other cell types due to Ca^2+^ responses induced by potassium depolarization (50 mM KCl for 2 s). In neuron 1 TRPV1 inhibitor capsazepine (capz, 10 μM) abolished NaHS induced Ca^2+^ transient; in neuron 2 capsazepine did not affect NaHS induced Ca^2+^ transient; in neuron 3 TRPA1 inhibitor HC 030031 (50 μM) abolished NaHS evoked response. **(B)** Graph indicating populations of capsaicin and NaHS sensitive TG neurons and their overlap. In a total of 251 imaged neurons (selected by their response to KCl) 104 (41%) were activated by NaHS (100 μM) and 107 (43%) by capsaicin (1 μM). In 59% of capsaicin sensitive cells NaHS also induced an increase of intracellular Ca^2+^ (63 cells from 107, gray area). **(C)** Percentage of cells responding to NaHS (100%) after inhibition of TRPV1 (Capz + NaHS) and TRPA1 (HC 030031 + NaHS) receptors.

In order to explore the action of the H_2_S donor on intracellular Ca^2+^ level in a large population of isolated neurons, we applied capsaicin (1 μM) and NaHS (100 μM) for 2 s before and after inhibition of TRPV1 or TRPA1 receptors by capsazepine (10 μM) or HC 030031 (50 μM), respectively. The subsequent application of KCl (50 mM) was used to distinguish neurons from glial cells (Kilic et al., 2017). As shown in Figures 3A,B NaHS induced Ca^2+^ transients in 41% neurons (104 of 251 cells); 43% of neurons tested were also sensitive to capsaicin (107 cells; Figure 3B). In 59% of cells responding to capsaicin, NaHS also induced an increase of intracellular Ca^2+^ (63 of 107 cells). Inhibition of TRPV1 receptors by capsazepine abolished Ca^2+^ responses evoked by NaHS in 37% of cells, which responded to NaHS and in 63% of cells the increase of [Ca^2+^]_in_ evoked by NaHS was still observed (Figures 3A,C). After inhibition of TRPA1 receptors by HC 030031, 80% of cells still showed an increase of [Ca^2+^]_in_ by NaHS application and in 20% of cells the NaHS response was eliminated (Figures 3A,C). In summary, in this approach the action of NaHS was partially sensitive to TRPV1 and TRPA1 antagonists.

## Discussion

The main finding of our study indicates that H_2_S induces an increase of firing activity in rat TG nerve and this effect is mediated by activation of both TRPV1 and TRPA1 receptors. In somas of TG neurons NaHS caused two types of effects on capsaicin evoked currents. In a fraction of neurons, NaHS induced a transient initial increase of the current amplitude followed by a subsequent decrease of responses. In the other fraction of cells, NaHS induced a progressive decline of TRPV1 currents. Moreover, H_2_S when locally applied to TG neurons elicits inward currents which were inhibited by the TRPV1 antagonist capsazepine but was not sensitive to the inhibitor of the TRPA1 receptors HC 030031. Furthermore, NaHS generated Ca^2+^ transients in TG neurons which were prevented by the inhibitors of TRPV1 and TRPA1 receptors. We propose that both TRPV1 and TRPA1 receptors in peripheral nerve endings in meninges and in somas of sensory neurons are involved in the pro-nociceptive action of H_2_S in the trigeminovascular system.

### H_2_S Increases Firing in TG Nerve by Activation of TRPV1 and TRPA1 Receptors

The TG system is directly involved in sensory and nociceptive conductance and TG nerve firing is involved in pain initiation during migraine. TRPA1 and TRPV1 receptors are widely expressed in capsaicin-sensitive sensory nerves (Huang et al., 2012). Activation of TRPV1 and TRPA1 on meningeal nerve endings induces the release of vasoactive neuropeptides CGRP and substance P and contributes to different forms of headache including migraine (Giniatullin et al., 2008; Benemei et al., 2013). Our results demonstrate that the donor of H_2_S—NaHS directly increases firing in TG nerve and this effect is mainly mediated by activation of TRPV1 as capsazepine, TRPV1 antagonist, completely abolished the effect of NaHS. At the same time the TRPA1 antagonist HC 030031 partially prevented the increase of TG nerve firing indicating the involvement of TRPA1 receptors in the H_2_S effect.

A number of indirect studies indicate the activation of TRPV1 and TRPA1 in afferent endings by H_2_S. It was shown that NaHS similar to capsaicin induced the release of CGRP and substance P from sensory nerves in the airways of guinea pig which causes bronchoconstriction *in vivo* (Patacchini et al., 2004; Trevisani et al., 2005). NaHS evoked contractions of the urinary bladder by activation of capsaicin-sensitive afferents and release of sensory neuropeptides (Patacchini et al., 2004). In the guinea-pig and human colon H_2_S caused mucosal Cl^−^ secretion (Schicho et al., 2006) and increased afferent firing in rat intestinal mesenteric nerves by activation of TRPV1 in afferent endings (Lu et al., 2014). On the other hand activation of TRPA1 by NaHS in afferent nerve fibers was reported to mediate an increased cutaneous blood flow by the release of CGRP and substance P in the mouse ear model (Hajna et al., 2016), whereas vasodilatory effects of H_2_S were reduced in mice lacking TRPA1 receptors (Pozsgai et al., 2012).

### H_2_S Directly Activates TRPV1 Receptors and Increases Intracellular Ca^2+^ Concentrations in Isolated TG Neurons

The focal application of NaHS on TG neurons induced inward currents similar to capsaicin which were inhibited by the TRPV1 antagonist capsazepine but were not sensitive to the inhibitor of TRPA1 antagonist HC 030031, which indicates a direct activation of TRPV1 receptors by H_2_S. At the same time superfusion of TG neurons with NaHS induced a bidirectional effect on capsaicin induced currents. In 63% of neurons NaHS induced an increase of the current amplitude during first minutes with subsequent desensitization, which can be explained by the reduction of disulfide bonds by H_2_S. Indeed, the sulfhydryl redox agent DTT in our experiments prevented the facilitating effect of NaHS. The redox modulation of TRPV1 receptors is well-known and it was shown than DTT greatly potentiated both native and recombinant rat TRPV1 channels at extracellularly located sites (Susankova et al., 2006). H_2_S is known for its reducing action which is responsible for its effects on Ca^2+^-activated K^+^ channels and NMDA-receptors (Abe and Kimura, 1996; Sitdikova et al., 2010; Kimura, 2016). In 36% of cells a constant decrease of TRPV1 currents was observed during NaHS superfusion, which may be explained by the rapid desensitization of TRPV1 receptors. This suggestion is supported by the variability in desensitization of capsaicin responses between different cell fractions. Indeed, the existence of several pools of TRPV1 receptors with slow and fast kinetics and with distinct rates of desensitization have been reported in TG neurons (Akopian et al., 2007; Storti et al., 2015; Zakharov et al., 2015) which may be determined by their lipid environment, coupling to caveolin, functional states, including redox state or phosphorylation (Storti et al., 2015). Moreover, the kinetics of TRPV1-mediated current depends on the co-expression of TRPA1 channels, with likely involvement of intracellular Ca^2+^ or other intracellular messengers affecting TRPV1 receptor desensitization (Masuoka et al., 2017). Similarly to our findings, in DRG neurons NaHS directly induced inward currents which were inhibited by capsazepine and A784168 (Lu et al., 2014). Our results suggest that H_2_S is a putative agonist of TRPV1 receptors in somas of TG neurons.

Fluorescence studies demonstrate that H_2_S increased the intracellular Ca^2+^ level in 41% of TG neurons cells, however, only 59% of H_2_S-sensitive cells responded to capsaicin, which reflects variability of H_2_S molecular targets and co-expression of other types of calcium-permeable receptors in TG neurons. Thus, in DRG neurons, the TRPV1 current densities were significantly smaller in allyl isothiocyanate (AITC)-sensitive DRG neurons than in AITC-insensitive cells and spontaneous TRPA1 channel activity inhibited the TRPV1 channels via Ca^2+^ elevation (Masuoka et al., 2017). Indeed 37% of H_2_S induced Ca^2+^ transients were inhibited by capsazepine which indicates mainly activation of TRPV1 receptors. However, 20% of H_2_S induced Ca^2+^ transients were also abolished by HC 030031 indicating the activation of TRPA1 receptors in a small fraction of neurons, which was supported by a number of other studies. It was shown that in TG neurons application of NaHS increased [Ca^2+^]_in_ in 20–42% of capsaicin-sensitive neurons with close correspondence between neurons that responded to NaHS and to AITC (Hajna et al., 2016). NaHS also evoked inward currents in DRG neurons and in CHO cells expressing TRPA1 receptors, which were inhibited by a TRPA1 antagonist (Miyamoto et al., 2011; Andersson et al., 2012; Ogawa et al., 2012).

However, it should be noted that high concentrations of NaHS (1–10 mM) were used in those studies which may activate TRPA1 indirectly by formation of reactive oxygen species (Andersson et al., 2008). Moreover, the main mechanism of TRPA1 activation appears to be oxidation of reactive cysteine residues whereas reducing agents induce an inhibition of TRPA1 (Macpherson et al., 2007). H_2_S being a reducing agent cannot induce direct oxidation of the thiol groups of proteins (Greiner et al., 2013). Recent studies report that polysulfides generated in NaHS and Na_2_S solutions or by the chemical interaction of H_2_S and NO are able to activate TRPA1 receptors (Hatakeyama et al., 2015; Kimura, 2016; Miyamoto et al., 2017). It appears possible therefore that the effective molecules which activate TRPA1 receptors in our and previous studies were polysulfides. However, in our experiments relatively low concentration of NaHS (100 μM, effectively 11 μM, see “Materials and Methods” Section) were used which probably is insufficient to generate sufficient amounts of polysulfides to activate TRPA1. Moreover, TRPV1 stimulation by H_2_S can cause Ca^2+^ dependent desensitization of TRPA1 receptor as TRPA1 is highly co-expressed with TRPV1 (Akopian et al., 2007; Palazzo et al., 2013).

The gating machinery of the TRPV1 receptor is complicated as different ligands are acting at distinct extra- and intracellular sites. The sites responsible for the reducing action of DTT are located at the extracellular part of the TRPV1 protein (Susankova et al., 2006) whereas capsaicin acts from the intracellular side (Gavva et al., 2004). Thus, the S512Y and Y511A point mutations at the intracellular part of the S3 segment were able to eliminate capsaicin sensitivity (Jordt and Julius, 2002). As the effect of NaHS was inhibited by capsazepine, a competitive antagonist of the TRPV1 receptor with structural similarities to capsaicin (Pingle et al., 2007), we propose that H_2_S activates the gating of the TRPV1 receptor at the same locus as capsaicin. However, the exact mechanisms of H_2_S action on the TRPV1 receptor have to be determined in future experiments.

In summary, our data suggest that H_2_S induces pro-nociceptive firing in the peripheral part of the TG nerve through activation of TRPV1 and TRPA1 receptors. This is consistent with the ability of H_2_S to induce membrane currents and Ca^2+^ transients in cell bodies of TG neurons, which were mediated by TRPV1 and TRPA1 receptors. The endogenous H_2_S producing enzyme CBS is abundantly expressed in rat TG neurons (Feng et al., 2013) and it is known that inflammation upregulates CBS expression in TG neurons at both protein and mRNA levels (Miao et al., 2014). A similar increase of CBS expression was observed also in rat DRG neurons after streptozotocin (Zhang et al., 2013) and complete Freund adjuvant treatments (Qi et al., 2013). We suggest that up-regulation of CSE or CBS during migraine related neuro-inflammation in meninges generates H_2_S resulting in activation of pro-nociceptive TRPV1 and TRPA1 receptors. Associated release of neuropeptide CGRP from TRPV1/TRPA1 positive peptidergic nerve fibers should further support both neuronal sensitization (Giniatullin et al., 2008) and the long-lasting nociceptive firing underlying migraine pain. Therefore, targeting the CBS-H_2_S-TRP signaling in the trigemino-vascular system might represent a novel therapeutic strategy for alleviation of TG pain.

## Author Contributions

KK, AM, AY, GS: experimental work and data acquisition. KK, AM, AY, GS: data analysis and preparation of figures. GS, RG, AH: study design/interpretation and drafting of manuscript. KK, AM, AY, AH, RG, GS: final approval of manuscript.

## Conflict of Interest Statement

The authors declare that the research was conducted in the absence of any commercial or financial relationships that could be construed as a potential conflict of interest.

## Abbreviations

CBS: cystathionine beta-synthase
CGRP: calcitonin gene-related peptide
CSE: cystathionine gamma-lyase
H_2_S: hydrogen sulfide
NaHS: sodium hydrosulfide
TG: trigeminal.

## References

[B1] Abe K. Kimura H. (1996). The possible role of hydrogen sulfide as an endogenous neuromodulator. J. Neurosci. 16, 1066–1071. 8558235 10.1523/JNEUROSCI.16-03-01066.1996 PMC6578817

[B2] Akopian A. N. Ruparel N. B. Jeske N. A. Hargreaves K. M. (2007). Transient receptor potential TRPA1 channel desensitization in sensory neurons is agonist dependent and regulated by TRPV1-directed internalization. J. Physiol. 583, 175–193. 10.1113/jphysiol.2007.133231 17584831 PMC2277224

[B3] Andersson D. A. Gentry C. Bevan S. (2012). TRPA1 has a key role in the somatic pro-nociceptive actions of hydrogen sulfide. PLoS One 7:e46917. 10.1371/journal.pone.0046917 23071662 PMC3469557

[B4] Andersson D. A. Gentry C. Moss S. Bevan S. (2008). Transient receptor potential A1 is a sensory receptor for multiple products of oxidative stress. J. Neurosci. 28, 2485–2494. 10.1523/JNEUROSCI.5369-07.2008 18322093 PMC2709206

[B5] Benemei S. De Cesaris F. Fusi C. Rossi E. Lupi C. Geppetti P. (2013). TRPA1 and other TRP channels in migraine. J. Headache Pain 14:71. 10.1186/1129-2377-14-71 23941062 PMC3844362

[B6] Bhatia M. Zhi L. Zhang H. Ng S.-W. Moore P. K. (2006). Role of substance P in hydrogen sulfide-induced pulmonary inflammation in mice. Am. J. Physiol. Lung Cell. Mol. Physiol. 291, L896–L904. 10.1152/ajplung.00053.2006 16798781

[B7] Deleon E. R. Stoy G. F. Olson K. R. (2012). Passive loss of hydrogen sulfide in biological experiments. Anal. Biochem. 421, 203–207. 10.1016/j.ab.2011.10.016 22056407

[B8] Distrutti E. Sediari L. Mencarelli A. Renga B. Orlandi S. Antonelli E.. (2006). Evidence that hydrogen sulfide exerts antinociceptive effects in the gastrointestinal tract by activating KATP channels. J. Pharmacol. Exp. Ther. 316, 325–335. 10.1124/jpet.105.091595 16192316

[B9] Feng X. Zhou Y.-L. Meng X. Qi F.-H. Chen W. Jiang X.. (2013). Hydrogen sulfide increases excitability through suppression of sustained potassium channel currents of rat trigeminal ganglion neurons. Mol. Pain 9:4. 10.1186/1744-8069-9-4 23413915 PMC3599800

[B11] Gavva N. R. Klionsky L. Qu Y. Shi L. Tamir R. Edenson S.. (2004). Molecular determinants of vanilloid sensitivity in TRPV1. J. Biol. Chem. 279, 20283–20295. 10.1074/jbc.M312577200 14996838

[B13] Gerasimova E. Lebedeva J. Yakovlev A. Zefirov A. Giniatullin R. A. Sitdikova G. (2015). Mechanisms of hydrogen sulfide (H2S) action on synaptic transmission at the mouse neuromuscular junction. Neuroscience 303, 577–585. 10.1016/j.neuroscience.2015.07.036 26192092

[B12] Gerasimova E. V. Yakovleva O. V. Zefirov A. L. Sitdikova G. F. Ze A. L. Sitdikova G. F. (2013). Role of ryanodine receptors in the effects of hydrogen sulfide on transmitter release from the frog motor nerve ending. Bull. Exp. Biol. Med. 155, 11–13. 10.1007/s10517-013-2067-7 23667860

[B14] Giniatullin R. Nistri A. Fabbretti E. (2008). Molecular mechanisms of sensitization of pain-transducing P2X3 receptors by the migraine mediators CGRP and NGF. Mol. Neurobiol. 37, 83–90. 10.1007/s12035-008-8020-5 18459072

[B15] Greiner R. Pálinkás Z. Bäsell K. Becher D. Antelmann H. Nagy P.. (2013). Polysulfides link H2S to protein thiol oxidation. Antioxid. Redox Signal. 19, 1749–1765. 10.1089/ars.2012.5041 23646934 PMC3837443

[B16] Hajna Z. Sághy E. Payrits M. Aubdool A. A. Szoke E. Pozsgai G.. (2016). Capsaicin-sensitive sensory nerves mediate the cellular and microvascular effects of H2S via TRPA1 receptor activation and neuropeptide release. J. Mol. Neurosci. 60, 157–170. 10.1007/s12031-016-0802-z 27525636

[B17] Hatakeyama Y. Takahashi K. Tominaga M. Kimura H. Ohta T. (2015). Polysulfide evokes acute pain through the activation of nociceptive TRPA1 in mouse sensory neurons. Mol. Pain 11:24. 10.1186/s12990-015-0023-4 25934637 PMC4428232

[B18] Huang D. Li S. Y. Dhaka A. Story G. M. Cao Y.-Q. Q. (2012). Expression of the transient receptor potential channels TRPV1, TRPA1 and TRPM8 in mouse trigeminal primary afferent neurons innervating the dura. Mol. Pain 8:66. 10.1186/1744-8069-8-66 22971321 PMC3489865

[B19] Jordt S.-E. Julius D. (2002). Molecular basis for species-specific sensitivity to “hot” chili peppers. Cell 108, 421–430. 10.1016/s0092-8674(02)00637-2 11853675

[B20] Kawabata A. Ishiki T. Nagasawa K. Yoshida S. Maeda Y. Takahashi T.. (2007). Hydrogen sulfide as a novel nociceptive messenger. Pain 132, 74–81. 10.1016/j.pain.2007.01.026 17346888

[B21] Kilic G. Guerrero-Toro C. Zakharov A. Vitale C. Gubert-Olive M. Koroleva K.. (2017). Serotonergic mechanisms of trigeminal meningeal nociception: implications for migraine pain. Neuropharmacology 116, 160–173. 10.1016/j.neuropharm.2016.12.024 28025094

[B22] Kimura H. (2016). Hydrogen polysulfide (H2Sn) signaling along with hydrogen sulfide (H2S) and nitric oxide (NO). J. Neural Transm. (Vienna) 123, 1235–1245. 10.1007/s00702-016-1600-z 27484215

[B23] Lu W. Li J. Gong L. Xu X. Han T. Ye Y.. (2014). H2S modulates duodenal motility in male rats via activating TRPV1 and KATP channels. Br. J. Pharmacol. 171, 1534–1550. 10.1111/bph.12562 24345161 PMC3954491

[B24] Macpherson L. J. Dubin A. E. Evans M. J. Marr F. Schultz P. G. Cravatt B. F.. (2007). Noxious compounds activate TRPA1 ion channels through covalent modification of cysteines. Nature 445, 541–545. 10.1038/nature05544 17237762

[B25] Masuoka T. Kudo M. Yamashita Y. Yoshida J. Imaizumi N. Muramatsu I.. (2017). TRPA1 channels modify TRPV1-mediated current responses in dorsal root ganglion neurons. Front. Physiol. 8:272. 10.3389/fphys.2017.00272 28515697 PMC5413491

[B26] Matsunami M. Tarui T. Mitani K. Nagasawa K. Fukushima O. Okubo K.. (2009). Luminal hydrogen sulfide plays a pronociceptive role in mouse colon. Gut 58, 751–761. 10.1136/gut.2007.144543 18852258

[B27] Miao X. Meng X. Wu G. Ju Z. Zhang H.-H. Hu S.. (2014). Upregulation of cystathionine-β-synthetase expression contributes to inflammatory pain in rat temporomandibular joint. Mol. Pain 10:9. 10.1186/1744-8069-10-9 24490955 PMC3917612

[B28] Miyamoto R. Koike S. Takano Y. Shibuya N. Kimura Y. Hanaoka K.. (2017). Polysulfides (H2Sn) produced from the interaction of hydrogen sulfide (H2S) and nitric oxide (NO) activate TRPA1 channels. Sci. Rep. 7:45995. 10.1038/srep45995 28378773 PMC5380989

[B29] Miyamoto R. Otsuguro K.-I. Ito S. (2011). Time- and concentration-dependent activation of TRPA1 by hydrogen sulfide in rat DRG neurons. Neurosci. Lett. 499, 137–142. 10.1016/j.neulet.2011.05.057 21658433

[B30] Mustafina A. N. Yakovlev A. V. Gaifullina A. S. Weiger T. M. Hermann A. Sitdikova G. F. (2015). Hydrogen sulfide induces hyperpolarization and decreases the exocytosis of secretory granules of rat GH3 pituitary tumor cells. Biochem. Biophys. Res. Commun. 465, 825–831. 10.1016/j.bbrc.2015.08.095 26319431

[B31] Ogawa H. Takahashi K. Miura S. Imagawa T. Saito S. Tominaga M.. (2012). H2S functions as a nociceptive messenger through transient receptor potential ankyrin 1 (TRPA1) activation. Neuroscience 218, 335–343. 10.1016/j.neuroscience.2012.05.044 22641084

[B32] Okubo K. Matsumura M. Kawaishi Y. Aoki Y. Matsunami M. Okawa Y.. (2012). Hydrogen sulfide-induced mechanical hyperalgesia and allodynia require activation of both Cav3.2 and TRPA1 channels in mice. Br. J. Pharmacol. 166, 1738–1743. 10.1111/j.1476-5381.2012.01886.x 22300342 PMC3419915

[B33] Palazzo E. Rossi F. de Novellis V. Maione S. (2013). Endogenous modulators of TRP channels. Curr. Top. Med. Chem. 13, 398–407. 10.2174/1568026611313030014 23432068

[B34] Patacchini R. Santicioli P. Giuliani S. Maggi C. A. (2004). Hydrogen sulfide (H2S) stimulates capsaicin-sensitive primary afferent neurons in the rat urinary bladder. Br. J. Pharmacol. 142, 31–34. 10.1038/sj.bjp.0705764 15051627 PMC1574935

[B35] Pingle S. C. Matta J. A. Ahern G. P. (2007). Capsaicin receptor: TRPV1 a promiscuous TRP channel. Handb. Exp. Pharmacol. 179, 155–171. 10.1007/978-3-540-34891-7_9 17217056

[B36] Pozsgai G. Hajna Z. Bagoly T. Boros M. Kemény Á. Materazzi S.. (2012). The role of transient receptor potential ankyrin 1 (TRPA1) receptor activation in hydrogen-sulphide-induced CGRP-release and vasodilation. Eur. J. Pharmacol. 689, 56–64. 10.1016/j.ejphar.2012.05.053 22721614

[B37] Qi F. Zhou Y. Xiao Y. Tao J. Gu J. Jiang X.. (2013). Promoter demethylation of cystathionine-β-synthetase gene contributes to inflammatory pain in rats. Pain 154, 34–45. 10.1016/j.pain.2012.07.031 23273102

[B38] Qu R. Tao J. Wang Y. Zhou Y. Wu G. Xiao Y.. (2013). Neonatal colonic inflammation sensitizes voltage-gated Na^+^ channels via upregulation of cystathionine β-synthetase expression in rat primary sensory neurons. Am. J. Physiol. Gastrointest. Liver Physiol. 304, G763–G772. 10.1152/ajpgi.00466.2012 23449670

[B39] Renga B. (2011). Hydrogen sulfide generation in mammals: the molecular biology of cystathionine-β- synthase (CBS) and cystathionine-γ-lyase (CSE). Inflamm. Allergy Drug Targets 10, 85–91. 10.2174/187152811794776286 21275900

[B40] Schicho R. Krueger D. Zeller F. Von Weyhern C. W. H. Frieling T. Kimura H.. (2006). Hydrogen sulfide is a novel prosecretory neuromodulator in the guinea-pig and human colon. Gastroenterology 131, 1542–1552. 10.1053/j.gastro.2006.08.035 17101327

[B41] Shatillo A. Koroleva K. Giniatullina R. Naumenko N. Slastnikova A. A. Aliev R. R.. (2013). Cortical spreading depression induces oxidative stress in the trigeminal nociceptive system. Neuroscience 253, 341–349. 10.1016/j.neuroscience.2013.09.002 24036374

[B42] Sitdikova G. F. Fuchs R. Kainz V. Weiger T. M. Hermann A. (2014). Phosphorylation of BK channels modulates the sensitivity to hydrogen sulfide (H2S). Front. Physiol. 5:431. 10.3389/fphys.2014.00431 25429270 PMC4228848

[B43] Sitdikova G. F. Khaertdinov N. N. Zefirov A. L. (2011). Role of calcium and potassium channels in effects of hydrogen sulfide on frog myocardial contractility. Bull. Exp. Biol. Med. 151, 163–166. 10.1007/s10517-011-1280-5 22238741

[B44] Sitdikova G. F. Weiger T. M. Hermann A. (2010). Hydrogen sulfide increases calcium-activated potassium (BK) channel activity of rat pituitary tumor cells. Pflugers Arch. 459, 389–397. 10.1007/s00424-009-0737-0 19802723

[B45] Storti B. Di Rienzo C. Cardarelli F. Bizzarri R. Beltram F. (2015). Unveiling TRPV1 spatio-temporal organization in live cell membranes. PLoS One 10:e0116900. 10.1371/journal.pone.0116900 25764349 PMC4357434

[B46] Susankova K. Tousova K. Vyklicky L. Teisinger J. Vlachova V. (2006). Reducing and oxidizing agents sensitize heat-activated vanilloid receptor (TRPV1) current. Mol. Pharmacol. 70, 383–394. 10.1124/mol.106.023069 16614139

[B47] Tang G. Wu L. Liang W. Wang R. (2005). Direct stimulation of K(ATP) channels by exogenous and endogenous hydrogen sulfide in vascular smooth muscle cells. Mol. Pharmacol. 68, 1757–1764. 10.1124/mol.105.017467 16150926

[B48] Trevisani M. Patacchini R. Nicoletti P. Gatti R. Gazzieri D. Lissi N.. (2005). Hydrogen sulfide causes vanilloid receptor 1-mediated neurogenic inflammation in the airways. Br. J. Pharmacol. 145, 1123–1131. 10.1038/sj.bjp.0706277 15937520 PMC1576227

[B49] Xu G.-Y. Winston J. H. Shenoy M. Zhou S. Chen J. D. Z. Pasricha P. J. (2009). The endogenous hydrogen sulfide producing enzyme cystathionine-β synthase contributes to visceral hypersensitivity in a rat model of irritable bowel syndrome. Mol. Pain 5:44. 10.1186/1744-8069-5-44 19660142 PMC2731739

[B50] Yakovlev A. V. Kurmasheva E. D. Giniatullin R. Khalilov I. Sitdikova G. F. (2017). Hydrogen sulfide inhibits giant depolarizing potentials and abolishes epileptiform activity of neonatal rat hippocampal slices. Neuroscience 340, 153–165. 10.1016/j.neuroscience.2016.10.051 27984177

[B51] Zakharov A. Vitale C. Kilic G. Koroleva K. Fayuk D. Shelukhina I.. (2015). Hunting for origins of migraine pain: cluster analysis of spontaneous and capsaicin-induced firing in meningeal trigeminal nerve fibers. Front. Cell. Neurosci. 9:287. 10.3389/fncel.2015.00287 26283923 PMC4516892

[B52] Zhang H.-H. Hu J. Zhou Y.-L. Hu S. Wang Y.-M. Chen W.. (2013). Promoted interaction of nuclear factor-κB with demethylated cystathionine-β-synthetase gene contributes to gastric hypersensitivity in diabetic rats. J. Neurosci. 33, 9028–9038. 10.1523/JNEUROSCI.1068-13.2013 23699514 PMC6705038

[B53] Zhu L. Zhao L. Qu R. Zhu H.-Y. Wang Y. Jiang X.. (2015). Adrenergic stimulation sensitizes TRPV1 through upregulation of cystathionine β-synthetase in a rat model of visceral hypersensitivity. Sci. Rep. 5:16109. 10.1038/srep16109 26527188 PMC4630780

